# Supplementary material for: Proximate ferromagnetic state in the Kitaev model material α-RuCl3
Source: Nat Commun. 2021 Jul 23;12:4512. doi: 10.1038/s41467-021-24722-4 (PMC8302668; doi:10.1038/s41467-021-24722-4)
Supplement: Supplementary file 1 — Supplementary Information [file 41467_2021_24722_MOESM1_ESM.pdf]

Supplementary Information for
Proximate ferromagnetic state in the Kitaev model material α-$RuCl_3$

H. Suzuki[1*], H. Liu[1*], J. Bertinshaw[1], K. Ueda[1], H. Kim[1,2,3], S. Laha[1], D. Weber[1], Z. Yang[1], L. Wang[1], H. Takahashi[1], K. Fürsich[1], M. Minola[1], B. V. Lotsch[1,4], B. J. Kim[1,2,3], H. Yavaş[5], M. Daghofer[6,7], J. Chaloupka[8,9], G. Khaliullin[1], H. Gretarsson[1,5], and B. Keimer[1*]

[1]*Max-Planck-Institut für Festkörperforschung, Heisenbergstraße 1, D-70569 Stuttgart, Germany*
[2]*Department of Physics, Pohang University of Science and Technology, Pohang 790-784, South Korea*
[3]*Center for Artificial Low Dimensional Electronic Systems, Institute for Basic Science (IBS), 77 Cheongam-Ro, Pohang 790-784, South Korea*
[4]*Department of Chemistry, University of Munich (LMU), Butenandtstraße 5-13 (Haus D), 81377 München, Germany*
[5]*Deutsches Elektronen-Synchrotron DESY, Notkestraße 85, D-22607 Hamburg, Germany*
[6]*Institute for Functional Matter and Quantum Technologies, University of Stuttgart, Pfaffenwaldring 57, 70550 Stuttgart, Germany*
[7]*Center for Integrated Quantum Science and Technology, University of Stuttgart, Pfaffenwaldring 57, 70550 Stuttgart, Germany*
[8]*Department of Condensed Matter Physics, Faculty of Science, Masaryk University, Kotlářská 2, 61137 Brno, Czech Republic and*
[9]*Central European Institute of Technology, Masaryk University, Kamenice 753/5, 62500 Brno, Czech Republic*

Supplementary Note 1: X-ray absorption spectrum and incident energy dependence of RIXS spectra

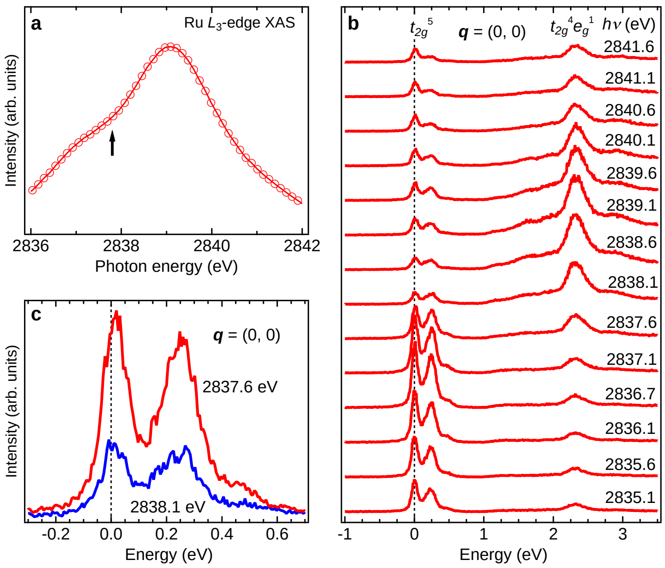

Fig. S1 X-ray absorption spectrum and RIXS spectra taken with different incident x-ray photon energies. a Ru L_3-edge x-ray absorption spectrum of $RuCl_3$ taken with π incident polarization. The arrow indicates 2837.8 eV used for the RIXS measurement in the main text. **b** Incident energy dependence of RIXS spectra at $\boldsymbol{q} = (0,0)$. **c** Expanded plot of low-energy RIXS spectra taken at 2837.6 (red) and 2838.1 eV (blue).

Figure S1a shows the x-ray absorption spectrum around the Ru L_3 edge taken with π incident polarization. 2837.8 eV used for the RIXS measurements in the main text is indicated by the arrow. It is located between a shoulder around 2837 eV corresponding to transitions to the t_{2g} orbitals and a peak around 2837 eV corresponding to transitions to the e_g orbitals. Figure S1b shows RIXS spectra taken with different incident photon energies around the Ru L_3 edge. With lower incident energies (< 2838 eV), the low-energy excitations within the t_{2g}^5 electron configurations are enhanced, whereas with higher incident energies (> 2838 eV) transitions to the $t_{2g}^4 e_g^1$ configurations are enhanced. Figure S1c compares the low-energy region of RIXS spectra taken at 2837.6 (red) and 2838.1 eV (blue), which are close to 2837.8 eV of the main text. Upon detuning from the t_{2g} resonance,

the quasi-elastic and $\widetilde{S}=3/2$ intensities weaken almost uniformly. This observation indicates that the quasi-elastic intensity is dominated by resonant magnetic scattering and contains little contributions from extrinsic non-resonant scattering channels, validating the intensity analysis presented in Fig. 5.

Supplementary Note 2: Determination of multiplet parameters from high-energy RIXS data

To quantitatively extract the multiplet parameters from the RIXS data shown in Fig. 2, we have diagonalized the single-ion Kanamori Hamiltonian for the d^5 electron configuration, which includes intra-ionic Coulomb interactions, spin-orbit coupling, and crystal fields. The Coulomb interactions are expressed using the intra-orbital interaction U, inter-orbital interaction U', and the Hund's-rule coupling J_H:

$$\begin{aligned} H_C &= U\sum_m n_{m\uparrow}n_{m\downarrow} + U'\sum_{m\neq m'} n_{m\uparrow}n_{m'\downarrow} \\ &+ (U'-J_H)\sum_{m<m',\sigma} n_{m\sigma}n_{m'\sigma} - J_H\sum_{m\neq m'} d^\dagger_{m\uparrow}d_{m\downarrow}d^\dagger_{m'\downarrow}d_{m'\uparrow} \\ &+ J_H\sum_{m\neq m'} d^\dagger_{m\uparrow}d^\dagger_{m\downarrow}d_{m'\downarrow}d_{m'\uparrow}, \end{aligned} \tag{1}$$

where $d^\dagger_{m\uparrow}$ and $n_{m\uparrow}$ are the electron creation and number operators, respectively. We employ the widely used approximation $U'=U-2J_H$. The spin-orbit coupling H_{so} and cubic crystal field H_{cub} are included as follows:

$$H_{so} = \lambda\sum_i \vec{l}_i\cdot\vec{s}_i, \tag{2}$$

$$H_{cub} = 10Dq\cdot\left(\tfrac{3}{5}n_{e_g} - \tfrac{2}{5}n_{t_{2g}}\right). \tag{3}$$

Using the eigenfunctions of the above Hamiltonian, we computed the RIXS transition amplitudes from the ground state doublet to the excited states within the fast-collision approximation as detailed in Ref. [1].

Supplementary Note 3: Selection of the pseudospin Hamiltonian parameters

TABLE SI: **Representative pseudospin models in the vicinity of the optimal parameter set.**

Models	K	J	Γ	Γ'	J_3	$E_{\text{FM}}-E_{\text{ZZ}}$ (meV)
Optimal set	-5	-3	2.5	0.1	0.75	-0.12
Alternative 1	-5	-2.5	2.5	0	0.5	-0.13
Alternative 2	-5	-2.5	2.5	0	0.75	0.05
Ref. [2]	-5	-0.5	2.5	0	0.5	0.37

Using the exact diagonalization (ED) method, we have examined a large number of models, inspecting their ability to explain our RIXS data. Along this extensive work, we observed that only the models which locate a system close to the ferromagnetic (FM) phase boundary are consistent with the data. On the classical level, the ground state energies of FM and zigzag (ZZ) states differ by $E_{\text{FM}}-E_{\text{ZZ}}=d\cdot S^2$, where $S=1/2$ and

$$d = \frac{1}{2}\sqrt{\left(K-\frac{1}{2}\Gamma+\Gamma'\right)^2+2\Gamma^2} + \frac{1}{2}\left(K-\frac{1}{2}\Gamma\right) + (J+3J_3) - \frac{3}{2}\Gamma'. \tag{4}$$

The proximity to the FM state implies small d values, which puts an additional constraint on the possible variations of the exchange parameters within the ZZ ordered state. We actually found that the models with a small negative $d\lesssim 0$ best describe the data. To illustrate this useful empirical rule, we present here the ED results for the RIXS intensities and also the dynamical spin responses, calculated for four representative models given in Table SI. In addition to the optimal parameter set and the

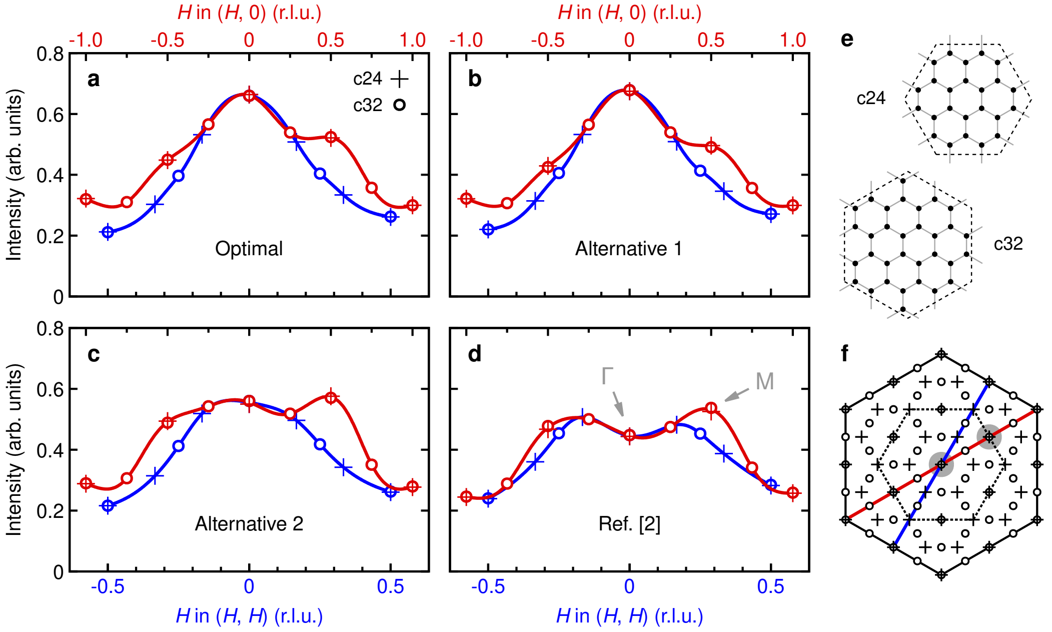

Fig. S2 Comparison of theoretical RIXS intensity at $T = 20\,\mathrm{K}$ for various parameter sets. a Theoretical RIXS intensity obtained for the optimal exchange parameters used in the main text: $(K, J, \Gamma, \Gamma', J_3) = (-5, -3, 2.5, 0.1, 0.75)$ meV. We have shown the intensities for the two scattering geometries corresponding to the $(H, 0)$ path (red) and the (H, H) path (blue). This panel thus reproduces selected data of Fig. 5. **b** The same for the parameter set $(K, J, \Gamma, \Gamma', J_3) = (-5, -2.5, 2.5, 0, 0.5)$ meV characterized by a similar proximity to the FM phase. **c** The same for the parameter set of panel **b** but with enhanced $J_3 = 0.75$ meV making the zigzag correlations more robust. **d** The same for the parameter set of Ref. [2]: $(K, J, \Gamma, \Gamma', J_3) = (-5, -0.5, 2.5, 0, 0.5)$ meV, again showing pronounced zigzag correlations at $T = 20\,\mathrm{K}$. **e** Hexagon-shaped clusters of 24 and 32 sites that were utilized in the numerical calculations to periodically cover the honeycomb lattice. **f** Accessible momenta for the clusters shown in panel **e** and the two cuts through the Brillouin zone explored by RIXS. Gray shading highlights the characteristic momenta for pseudospin correlations: the $\boldsymbol{q} = \Gamma$ point (center) hosts FM correlations while the zigzag correlations are associated with the $\boldsymbol{q} = M$ point (upper right).

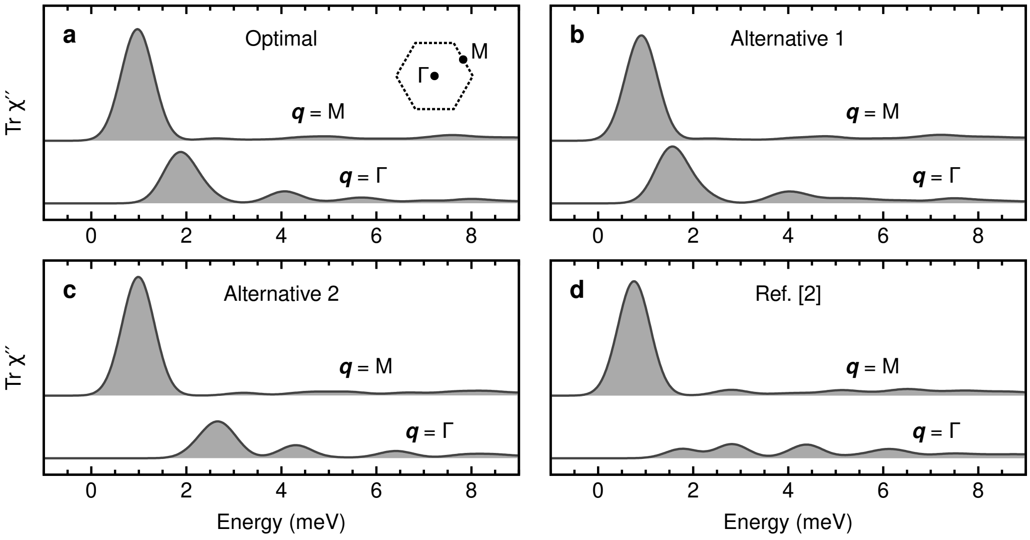

Fig. S3 Dynamical response of pseudospin models. a-d The trace of the pseudospin susceptibility at the two most relevant $\boldsymbol{q}$ points (imaginary part is plotted). The parameter sets considered here are identical to those for panels **a-d** of Fig. S2. The pseudospin susceptibility was calculated by exact diagonalization using the 24-site cluster and broadened by a Gaussian profile.

model by Winter et al. (Ref. [2]) discussed in the main text, we consider alternative parameter sets 1 and 2 in the vicinity of the optimal set. The ED calculations confirm that all models have the zigzag ground state with the correct moment direction. For the first two models with $E_{\mathrm{FM}} - E_{\mathrm{ZZ}} < 0$, the classical ground state would have been FM, and thus the ZZ order in these models is stabilized by quantum effects, which shift the actual ZZ/FM phase boundary from that found in the classical approximation. The last two models give the ZZ order already on a classical level.

We first look at the $\boldsymbol{q}$ dependence of the theoretical RIXS intensity (Fig. S2). Panels **a-d** correspond to the models indicated in the inset of each panel. The calculations were performed for hexagonal clusters of 24 and 32 sites shown in panel **e**. The accessible momenta for these clusters and the momentum paths studied are shown in panel **f**. The global intensity maximum in panels **a** and **b** is located at the Γ point as observed in RIXS, whereas it is located at the M point (Bragg wavevector of the zigzag order) in panels **c** and **d**. We recall that, when treated classically, the optimal set and alternative set 1 would have the ferromagnetic ground state, while the alternative set 2 and the set of Ref. [2] have more robust ZZ ground state (Table SI). This comparison demonstrates that one needs to stay very close to the FM phase to have sufficiently pronounced $\boldsymbol{q} \sim 0$ correlations at 20 K as observed in RIXS. Underlying picture is that as soon as the quantum effects, favoring the ZZ order over competing FM state, are weakened by thermal fluctuations, spectral weight moves to $\boldsymbol{q} \sim \Gamma$ area hosting FM correlations.

However, there is some freedom in choosing the model parameters if the proximity to FM is kept: cf. panels **a** and **b** representing two slightly different sets but with very similar values of $d \sim -0.5$ meV. Fine selecion between them can be made by comparing the corresponding dynamical spin response functions with inelastic neutron scattering (INS) data. In particular, the recent INS data that have sufficient resolution to see the actual energy profiles [3] are helpful to narrow down the parameter window. Figure S3 shows dynamical pseudospin correlations captured by the susceptibility tensor $\chi_{\alpha\beta}(\boldsymbol{q},\omega) = \langle \widetilde{S}^{\alpha}_{\boldsymbol{q}} \widetilde{S}^{\beta}_{-\boldsymbol{q}} \rangle_{\omega}$ whose energy-integrated components determined the RIXS spectral weights above. We plot the imaginary part of its trace to show some coordinate-free characteristic profiles. The parameter sets for panels **a-d** are the same as those in Fig. S2. If one compares the optimal set (**a**) and alternative set 1 (**b**), the position of the peak at $\boldsymbol{q} = \Gamma$ favors the former as it is more consistent with the INS data that shows a sharp magnon peak around 2 meV [3]. The alternative set 2 (**c**) is not consistent with RIXS (although the agreement with INS is decent), and the set of Ref. [2] (**d**) with small J completely misses the well-defined magnon peak observed in INS.

Supplementary Note 4: Paramagnetic susceptibility and trigonal crystal field

Here we estimate the trigonal crystal field in $RuCl_3$ from the magnetic susceptibility anisotropy in single crystals. We have measured the paramagnetic susceptibility up to 370 K, and fit the data using the Curie-Weiss law:

$$\chi = \frac{C}{T - \Theta} + \chi_0. \tag{5}$$

The inverse magnetic susceptibilities $1/(\chi - \chi_0)$ for in-plane (H // ab) and out-of-plane (H // c) magnetic fields are shown in Fig. S4a and b, respectively, along with the results of fits to the Curie-Weiss law (red curves). The deviations from Curie-Weiss behavior can be attributed to short-range correlations present well above the magnetic ordering temperature (see Fig. 5f and related discussion).

The paramagnetic Curie temperatures for both ab-plane and c-axis magnetic field directions are positive, $\Theta_{ab} \simeq 50$ K and $\Theta_c \simeq 30$ K, respectively. These values are roughly consistent with the exchange constants obtained from the RIXS data ($K = -5$ meV, $J = -3$ meV, $J_3 = 0.75$, $\Gamma + 2\Gamma' = 2.7$ meV):

$$\begin{aligned} \Theta_{ab} &= -\tfrac{3}{4}\left[J + J_3 + \tfrac{1}{3}K - \tfrac{1}{3}(\Gamma + 2\Gamma')\right] \sim 40\,\mathrm{K}, \\ \Theta_{c} &= -\tfrac{3}{4}\left[J + J_3 + \tfrac{1}{3}K + \tfrac{2}{3}(\Gamma + 2\Gamma')\right] \sim 20\,\mathrm{K}. \end{aligned} \tag{6}$$

The high temperature slopes in $1/(\chi - \chi_0)$ plots are clearly different, resulting in anisotropic Curie constants ($C^{ab} \sim 3C^{c}$) and g-factors; we find $|g_{ab}| \simeq 2.53$ and $|g_c| \simeq 1.56$, with an average value of $|g| \simeq 2.2$. Even though the maximum temperature ~ 370 K might be not high enough to access the true Curie-Weiss behavior, it is evident that the g-factor anisotropy is robust in $RuCl_3$. A similar anisotropy ($|g_{ab}| \simeq 2.3$ and $|g_c| \simeq 1.3$) has been deduced from the analysis of low-temperature magnetization data [4], using similar exchange constants as above. Overall, the magnetic anisotropy in $RuCl_3$ originates from a combination of single-ion effects (i.e., the g-factors) and the exchange anisotropy (i.e., the Γ and, to a lesser extent, Γ' terms), as expected for spin-1/2 systems with a layered crystal structure.

The g-factors are sensitive to the orbital content of the ground state wavefunction, and thus contain useful information on the strength of the trigonal crystal field [5] which splits the t_{2g} electron level into an a_{1g} singlet and an e_g' doublet by $\Delta =$

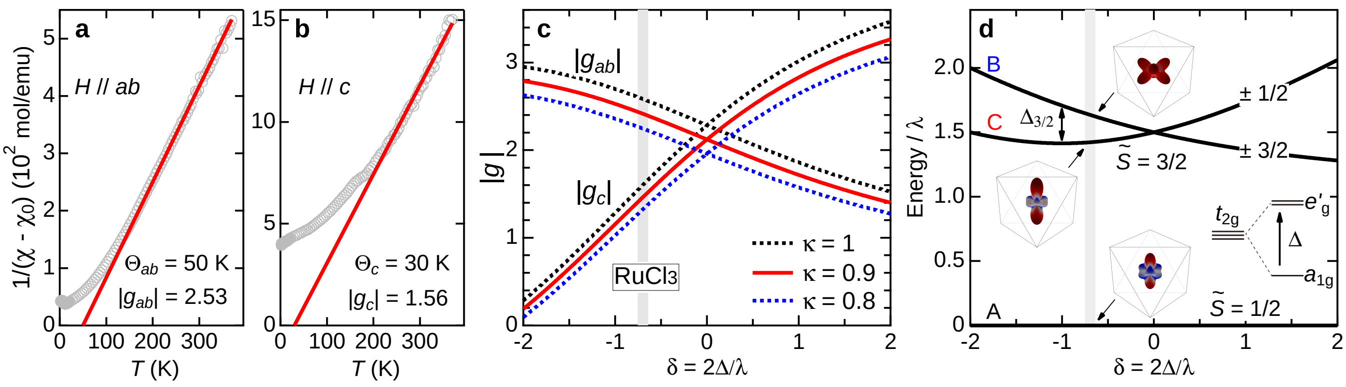

Fig. S4 Estimation of trigonal crystal field based on paramagnetic susceptibility. a, b In-plane (H // ab) and out-of-plane (H // c) inverse magnetic susceptibility $1/(\chi-\chi_0)$. The red lines show the fits to the Curie-Weiss law [Eq. (5)] with parameters $\chi_0^{ab}=1.0\times10^{-4}$ emu/mol and $C^{ab}=0.60$ emu·K/mol, and $\chi_0^{c}=1.0\times10^{-3}$ and $C^{c}=0.229$ emu·K/mol. The resulting g-factors are $|g_{ab}|=2.53$ and $|g_c|=1.56$. **c** The g-factors of the pseudospin $\widetilde{S}=1/2$ as a function of the trigonal field parameter $\delta=2\Delta/\lambda$ [Eq. (8)], at the covalency factors of $\kappa=1$ (dotted black), $\kappa=0.9$ (solid red), and $\kappa=0.8$ (dotted blue). The grey vertical line indicates the trigonal field $\delta\sim-0.7$ consistent with the g-factors in $RuCl_3$. **d** Energy levels of the pseudospin $\widetilde{S}=3/2$ states. Away from cubic limit ($\Delta=0$), they split into two doublets separated by $\Delta_{3/2}$. The $\widetilde{S}=1/2$ and 3/2 wavefunctions for $\delta=-0.7$ are depicted. The red and blue colors represent the spin components $S=1/2$ and $-1/2$ along the trigonal axis, respectively. Inset: t_{2g} orbital splitting Δ under the trigonal crystal field (electron picture).

$E(e_g')-E(a_{1g})$ (see the inset of Fig. S4d). The trigonal field Δ and spin-orbit coupling λ quantify the ground state pseudospin $\widetilde{S}=1/2$ wavefunctions of the Ru^{3+} ion as follows:

$$\left|+\frac{1}{2}\right\rangle = +\sin\theta|0,\uparrow\rangle-\cos\theta|+1,\downarrow\rangle\ ,$$
$$\left|-\frac{1}{2}\right\rangle = -\sin\theta|0,\downarrow\rangle+\cos\theta|-1,\uparrow\rangle\ . \quad (7)$$

The angle $0\le\theta\le\pi/2$ is given by $\tan 2\theta = 2\sqrt{2}/(1+\delta)$, where $\delta=2\Delta/\lambda$. The wavefunctions above are written in the basis of $|L_Z,S_Z\rangle$ using the trigonal field quantization axis. The relative phases of the Kramers partners $|\pm\frac{1}{2}\rangle$ are chosen such that the g-factor is isotropic in the cubic limit, $g=-2$; its negative sign emphasizes the presence of a large orbital contribution to the magnetic moment. The latter is given by $\boldsymbol{M}=(2\boldsymbol{S}-\kappa\boldsymbol{L})$, where $\boldsymbol{L}$ is the effective orbital moment of the t_{2g} level and κ is the covalency reduction factor [5].

The wavefunctions in Eq. (7) assume a pure t_{2g}^5 configuration in the ground state. The admixture of $t_{2g}^4e_g$ configurations by electron interactions results in a correction Δg to the g-factors [6]. Including this effect, one obtains

$$g_{ab} = -[1-\cos 2\theta+\sqrt{2}\kappa\sin 2\theta+\Delta g]\ ,$$
$$g_c = -[\kappa+(2+\kappa)\cos 2\theta+\Delta g]\ . \quad (8)$$

Adopting the results of Ref. [6] and assuming the relation $8B\simeq J_H$ between the Racah parameter B and Hund's coupling J_H, we obtain $\Delta g\simeq 2\kappa J_H/10Dq$. Since the $t_{2g}^4e_g$ configuration energy is rather low in $RuCl_3$, this correction is sizeable; using $J_H=0.34$ eV and $10Dq=2.4$ eV as measured in this work, we find $\Delta g\simeq 0.28\kappa$.

Figure S4c shows the g-factors as a function of the trigonal field parameter δ, for several values of the covalency factor κ. The g-factors $|g_{ab}|\simeq 2.53$ and $|g_c|\simeq 1.56$ obtained from the Curie-Weiss fits are well reproduced by the above equations at $\delta=-0.70$ and $\kappa=0.97$. We find that $|g_{ab}|\simeq 2.3$ and $|g_c|\simeq 1.3$ of Ref. [4] result in a nearly identical trigonal field parameter $\delta=-0.75$, and smaller $\kappa=0.82$. With $\lambda=0.15$ eV obtained from our RIXS data (see Fig. 2), $\delta\sim-0.7$ yields the trigonal splitting of $\Delta\sim-50$ meV (trigonal elongation within a point charge model). This splitting is roughly consistent with the quantum chemistry calculations of Ref. [7] (~-70 meV), and somewhat larger than that reported in Ref. [8] (-12±10 meV). $\Delta\sim-50$ meV is rather small compared to the spin-orbit coupling λ; this implies that the orbital magnetism, which is responsible for the bond-dependent Kitaev interactions, remains unquenched.

Apart from modification of the ground state wavefunctions, the trigonal crystal field splits the spin-orbit $\widetilde{S} = 3/2$ excitation into two doublets, labeled by B and C in Fig. S4d, by an amount $\Delta_{3/2} = \frac{\lambda}{4}[\sqrt{8+(1+\delta)^2} - 3 + \delta]$ [9]. With $\delta = -0.70$, we obtain the $\widetilde{S} = 3/2$ quartet splitting of $\Delta_{3/2} \sim 30$ meV. This splitting is much smaller than the current energy resolution of 0.1 eV, and is consistent with the absence of a clear splitting of the A_1 peaks in the RIXS spectra (see Fig. 3).

Supplementary Note 5: X-ray self-absorption correction

Due to the x-ray self-absorption, the scattering intensity observed by the spectrometer is reduced from the differential cross section. The reduction factor f is given by [10]:

$$f = 1 + \frac{\alpha_{\text{out}}(E_{\text{out}})}{\alpha_{\text{in}}(E_{\text{in}})} \cdot \frac{\sin(\theta)}{\sin(\chi)}, \tag{9}$$

where $\alpha_{\text{in}}(E_{\text{in}})$ $[\alpha_{\text{out}}(E_{\text{out}})]$ is the absorption coefficient for the incoming (outgoing) x-ray energy, light path, and polarization, and θ (χ) is the angle between the sample and the incoming (outgoing) x-rays. In our scattering geometry $\chi = \pi/2 - \theta$ holds. For the RIXS intensity analysis presented in Fig. 5 of the main text, we are concerned only with the low-energy excitations ($<$ 0.25 eV) created by the incident x-ray photons of 2837.8 eV. As shown in Fig. S1a, the change of x-ray absorption coefficient within this energy range is small, and it is also shown in Ref. [8] that the x-ray linear dichroism around the Ru L_3 edge is small. We have therefore taken the ratio $\frac{\alpha_{\text{out}}(E_{\text{out}})}{\alpha_{\text{in}}(E_{\text{in}})}$ to be unity and the correction coefficient depends only on the scattering geometry.

[1] Kim, B. J. & Khaliullin, G. Resonant inelastic x-ray scattering operators for t_{2g} orbital systems. *Phys. Rev. B* **96**, 085108 (2017).
[2] Winter, S. M. et al. Breakdown of magnons in a strongly spin-orbital coupled magnet. *Nat. Commun.* **8**(1), 1152 (2017).
[3] Balz, C. et al. Finite field regime for a quantum spin liquid in α-$RuCl_3$. *Phys. Rev. B* **100**, 060405(R) (2019).
[4] Winter, S. M., Riedl, K., Kaib, D., Coldea, R & Valentí, R. Probing α-$RuCl_3$ beyond magnetic order: Effects of temperature and magnetic field. *Phys. Rev. Lett.* **120**, 077203 (2018).
[5] Abragam, A. & Bleaney, B. *Electron Paramagnetic Resonance of Transition Ions*. Clarendon Press, Oxford, (1970).
[6] Thornley, J. H. M., Lustig, C. D., Owen, J & Thornley, J. H. M. The magnetic properties of $(IrX_6)^{2-}$ complexes. *J. Phys. C: Solid State Phys.* **1**(4), 1024–1037 (1968).
[7] Yadav, R. et al. Kitaev exchange and field-induced quantum spin-liquid states in honeycomb α-$RuCl_3$. *Sci. Rep.* **6**, 37925 (2016).
[8] Agrestini, S. et al. Electronically highly cubic conditions for Ru in α-$RuCl_3$. *Phys. Rev. B* **96**, 161107 (2017).
[9] Chaloupka, J. & Khaliullin, G. Magnetic anisotropy in the Kitaev model systems Na_2IrO_3 and $RuCl_3$. *Phys. Rev. B* **94**, 064435 (2016).
[10] Minola, M. et al. Collective nature of spin excitations in superconducting cuprates probed by resonant inelastic x-ray scattering. *Phys. Rev. Lett.* **114**, 217003 (2015).
